# TBX3 transfection and nodal signal pathway inhibition promote differentiation of adipose mesenchymal stem cell to cardiac pacemaker-like cells

**DOI:** 10.1186/s13287-024-03760-x

**Published:** 2024-05-22

**Authors:** Faris Basalamah, Ismail Hadisoebroto Dilogo, Sunu Budhi Raharjo, Muchtaruddin Mansyur, Nuryati Chairani Siregar, Nurhadi Ibrahim, Budi Yuli Setianto, Yoga Yuniadi

**Affiliations:** 1 Doctoral Program in Medical Science, Faculty of Medicine, Universitas Indonesia, Jakarta, 10430 Indonesia; 2 Faculty of Medicine and Health, University Muhammadiyah Jakarta, Banten, 15419 Indonesia; 3 Department of Cardiology and Vascular Medicine, Faculty of Medicine, Universitas Indonesia-National Heart Center Harapan Kita, Jakarta, 10430 Indonesia; 4 Department of Orthopedic and Traumatology, Faculty of Medicine, Universitas Indonesia-Dr Cipto Mangunkusumo National General Hospital, Jakarta, 10430 Indonesia; 5 Stem Cell and Tissue Engineering Research Cluster, Indonesia Medical Education and Research Institute, Faculty of Medicine, Universitas Indonesia, Jakarta, 10430 Indonesia; 6 Stem Cell Medical Technology Integrated Service Unit, Dr Cipto Mangunkusumo National General Hospital, Jakarta, 10430 Indonesia; 7 Department of Community Medicine, Faculty of Medicine, Universitas Indonesia, Jakarta, 10430 Indonesia; 8 Department of Anatomical Pathology, Faculty of Medicine, Universitas Indonesia, Jakarta, 10430 Indonesia; 9 Department of Medical Physiology and Biophysics, Faculty of Medicine, Universitas Indonesia, Jakarta, 10430 Indonesia; 10 Neuroscience and Brain Development Research Cluster, Indonesian Medical Education and Researsch Institute, Faculty of Medicine, Universitas Indonesia, Jakarta, 10430 Indonesia; 11 Department of Cardiology and Vascular Medicine, Faculty of Medicine, Health and Nursing, Universitas Gajah Mada, Yogyakarta, 55281 Indonesia; 12 Department of Cardiology and Vascular Medicine, Faculty of Medicine, Universitas Indonesia-RS Jantung Dan Pembuluh Darah Harapan Kita, Jakarta, 10420 Indonesia

**Keywords:** Mesenchymal stem cells, Cardiac pacemaker-like cells, TBX3, Nodal signal pathway, Action potential

## Abstract

**Background:**

Mesenchymal stem cells (MSCs) are known as one of the best candidate cells to produce cardiac pacemaker-like cells (CPLCs). Upregulation of TBX3 transcription factor and inhibition of the nodal signal pathway have a significant role in the formation of cardiac pacemaker cells such as sinoatrial and atrioventricular nodes, which initiate the heartbeat and control the rhythm of heart contractions. This study aimed to confirm the effects of transfection of TBX3 transcription factor and inhibition of the nodal signal pathway on differentiating adipose-derived MSCs (AD-MSCs) to CPLCs. AD-MSCs were characterized using flow cytometry and three-lineage differentiation staining.

**Methods:**

The transfection of TBX3 plasmid was carried out using lipofectamine, and inhibition of the nodal signal pathway was done using the small-molecule SB431542. The morphology of the cells was observed using a light microscope. Pacemaker-specific markers, including TBX3, Cx30, HCN4, HCN1, HCN3, and KCNN4, were evaluated using the qRT-PCR method. For protein level, TBX3 and Cx30 were evaluated using ELISA and immunofluorescence staining. The electrophysiology of cells was evaluated using a patch clamp.

**Results:**

The TBX3 expression in the TBX3, SM, and TBX + SM groups significantly higher (*p* < 0.05) compared to the control group and cardiomyocytes. The expression of Cx40 and Cx43 genes were lower in TBX3, SM, TBX + SM groups. In contrast, Cx30 gene showed higher expression in TBX3 group. The expression HCN1, HCN3, and HCN4 genes are higher in TBX3 group.

**Conclusion:**

The transfection of TBX3 and inhibition of the nodal signal pathway by small-molecule SB431542 enhanced differentiation of AD-MSCs to CPLCs.

## Introduction

Mesenchymal stem cells (MSCs) have been widely studied, both preclinically and clinically, in the treatment of ischemic heart disease and heart failure [1]. MSCs have also been shown to improve atrioventricular conduction in animal models [2]. Moreover, MSCs can be isolated from adipose tissue [3], as they are known to differentiate into both cardiomyocytes and pacemaker-like cells [4]. Several methods can be used to differentiate MSCs into pacemaker-like cells, including the use of genetically engineered MSCs [4]. Genetic engineering methods can be carried out by transfection, which involves inserting a gene into the cell that is integrated into the cell and can be expressed in the form of a protein [4].

TBX3 plays a role in the development and operation of the cardiac conduction system [5]. It has the function to repress atrial myocardium genes such as Cx40, Cx43, and ANF to direct cell development toward pacemaker cells [6]. TBX3 overexpression in the atrial myocardium may even lead to the development of ectopic pacemakers, based on studies of overexpression in TBX3 mutant mice [7]. A study also showed that pacemakers originate from TBX3-positive cells in early heart organ development [8]. In several previous studies, by inducing TBX3 in pluripotent stem cells, the stem cells were significantly induced to differentiate into pacemaker-like cells and express markers such as SHOX2 and NKX2.5. The results of this study indicated that manipulation of TBX3 in stem cells at the pan-cardiomyocyte stage can lead the cells to differentiate into pacemaker-like cells that may be used for therapy [9]. In another study examining the role of TBX3 in forming the atrioventricular conduction system, it was shown that in the absence of TBX3 gene expression in mouse embryonic development, pacemaker tissue is not formed, which results in death on days 12–15 of embryonic age. By analyzing the expression of other genes such as Cx43, Cx40, TBX18, and TBX20, the study also demonstrated that TBX3 can repress differentiation into the myocardium and enhance the phenotype of the cardiac conduction system [10].

The development of pacemaker cells is also affected by inhibition of the nodal protein signaling pathway [11]. Nodal signaling pathways are very important in determining embryonic development patterns in vertebrates [12]. Nodal itself is a molecule that acts as a conduction signal, is part of the TGF-protein family, and has an important role in the specification of mesoderm institutions and the determination of anterior–posterior symmetry and the right-left axis in the gastrulation phase in embryonic development [13, 14]. The PITX2 protein is one of the targets of the nodal protein signaling pathway, which is expressed asymmetrically during gastrulation [15], but the PITX2 gene plays a role in cardiac development and right-left heart symmetry [16]. In another study, pacemaker-like cells were differentiated by inhibiting the PITX2 gene, which functions in the inhibition of sinoatrial (SA) nodes formation in embryonic development [17]. PITX2 has been shown to downregulate the SHOX2 transcription factor, which plays a role in increasing TBX3 expression [18, 19]. Therefore, PITX2 inhibition can indirectly increase TBX3 expression, which can direct stem cell differentiation into pacemaker-like cells.

Until now, there is still a lack of evidence that demonstrates TBX3 transcription factor and inhibition nodal signal pathway can regulate differentiation of MSCs into cardiac pacemaker-like cells (CPLCs). In this study, MSCs from adipose tissue were differentiated into pacemaker-like cells by transfection of the pcDNA TBX3 plasmid and inhibition nodal signal pathway using small-molecule SB431542. After differentiation, the cells were then analyzed for pacemaker cells markers using qRT-PCR, ELISA, immunofluorescence, and patch clamp.

## Material and methods

### MSC isolation and culture

AD-MSCs of a single healthy subject were obtained from the cell bank of the Stem Cells and Tissue Engineering (SCTE) Laboratory, IMERI, Faculty of Medicine, Universitas Indonesia. The study was approved by Ethics Committee of the Faculty of Medicine, Universitas Indonesia. AD-MSCs was isolated from adult adipose tissue with the liposuction method referred to Pawitan's study [20]. In brief, the isolation was performed by simple lipoaspirate washing using a fine mesh stainless steel filter.

The AD-MSCs were cultured at 37 °C and 5% CO_2_ using a complete medium for cell propagation referred to Pawitan's study [20]. The complete medium included penicillin–streptomycin 1% (final concentration 100 U/mL) (Gibco, 15140122), amphotericin-B 1% (final concentration 2500 ng/mL) (Gibco, 15290026), glutaMAX 1% (Gibco, 15290026), heparin 1%, platelet concentrate 10% (Indonesian Red Cross), and alpha modification of Eagle's medium (α-MEM; basal cell culture medium) (Gibco, 51200038). The culture medium was changed every 2–3 days until the culture reached 80–90% confluence and was ready to be harvested. For the all treatment groups, AD-MSCs were seeded in culture plates with 12 wells at the density of 10.000 cells/cm^2^, and were differentiated when the culture reached 60–70% confluence. The treatment groups in this study were as follows:

- AD-MSCs control group

AD-MSCs cultured in medium complete for MSC without differentiated.

- Cardiomyocyte group (CAR)

AD-MSCs which had reached 60–70% confluence were differentiated using a PSC Cardiomyocyte Differentiation Kit (Gibco, A2921201) that consist of medium A, medium B, and maintenance medium. On day 1 and day 2, cells were cultured with medium A. On day 3 to day 5, cells were cultured with medium B. After that, cells were cultured with maintenance medium until day 15.

- TBX3 transfection group (TBX3)

AD-MSCs which had reached 60–70% confluence were differentiated using a PSC Cardiomyocyte Differentiation Kit (Gibco, A2921201). On day 3, differentiation culture was transfected with 1000 ng TBX3 cDNA ORF Human (Sinobiological, HG18335-UT) using Lipofectamine™ 2000 Transfection Reagent (Invitrogen, 11668019). The plasmid vector was pCMV3-untagged with restriction site KpnI + XbaI (6.1 kb + 2.17 kb).

- Small-molecules group (SM)

AD-MSCS differentiation culture with 2 µM/mL small-molecule SB431542 (Tocris, 301836-41-9) added.

- TBX3 transfection + Small molecules group (TBX3 + SM)

AD-MSCS differentiation culture transfected with 1000 ng pcDNA using lipofectamine and 2 µM/mL small-molecule SB431542.

## MSC characterization: flowcytometry

For surface marker analyses, the cultured cells were stained with a Human MSC Analysis Kit (BD Biosciences, 562245) according to manufacturer's instructions. The stained cells were then loaded into a flowcytometer (FACS AriaIII, BD Biosciences) to confirm the purity of CD73, CD90, and CD105 positive cells. For analysis of MSC cell surface markers, the antibodies used were PE hMSC cocktail positive and negative for isotypes and stains and a Human MSC Analysis Kit. The isotype and stain tubes each contained 100 µL of sample and 2 µL of antibody. Then, the samples were incubated for 20–30 min in the dark. After that, 100 µL of PBS was added to each tube.

## MSC characterization: three lineages differentiation

AD-MSCs were seeded in culture plates with 12 wells at the density of 25,000 cells/cm^2^. After the cells reached 60% confluence, the culture medium was changed to differentiation induction medium. Cells were cultured at 37 °C and 5% CO2. The differentiation medium (Gibco, A1007201, A1007001) was replaced every 2–3 days until the cells reached confluence and showed differentiation characteristics that could be observed using a microscope. The differentiated cells were then stained.

The first stage of the staining process was removing the cell medium, and then the cells were washed using PBS. Cells were fixed using 4% formaldehyde for 30 min at room temperature. After that, the formaldehyde was removed from the cells and the cells were rinsed using PBS. Cell staining was carried out specifically according to the results of differentiation. Osteocytes were stained by adding 2% Alizarin red pH 4.1–4.3 for 20 min at room temperature. Chondrocytes were stained by adding 1% Alcian blue dissolved in 0.1 HCl for 30 min at room temperature. Adipocytes were stained by adding 0.5% red o oil dissolved in 60% isopropanol for 2–5 min at room temperature. If the color intensity was considered sufficient, then rinsing was carried out using aquabides and the cells were captured using a microscope.

### RNA isolation and cDNA synthesis

Isolation of RNA from cultured cells was performed using Quick-RNA Miniprep Kit (Zymo Research, R1055) according to manufacturer's instructions. The initial stage of RNA isolation was started by washing with PBS twice. Cells were released from culture containers by trypsinization and centrifugation. The pellets formed were then processed to obtain pure RNA.

The isolated RNA with concentration 100 ng/μL was then converted into cDNA using the ReverTra Ace™ qPCR RT Master Mix with gDNA Remover (Toyobo, FSQ-301) according to manufacturer's instructions. The tools used for cDNA synthesis were a VeritiTM Thermal Cycler 96 well and Gradient PCR (Applied Biosystem) with conditions set at 42 °C for 30 min then 95 °C for 3 min.

### qRT-PCR analysis

Quantification and amplification of the target genes by qRT-PCR was carried out according to the procedure in the SensiFAST™ SYBR® Lo-ROX Kit (Bioline, BIO-94005) with the list of primers showed on Table 1. A total of 2 μL of cDNA synthesis with concentration 10 ng/μL was used as a template for the qRT-PCR reaction under the following conditions: enzyme activation at 95 °C for 3 min; denaturation at 95 °C for 1–3 s; annealing and elongation at 60 °C for > 20 s. The cycle threshold (CT) value was obtained from the cycle point when the amplification curve started the exponential phase. The CT values obtained were then processed using the Livak formula to determine the relative expression value of the target gene against the reference gene.

**Table 1:** List of primers for qRT-PCR analysis

No	Gene	Access code	Primer (5′–3′)
1	TBX3	NM_005996.4	F: GAGATGTTCTGGGCTGGATAAA R: CCATCCACCGAGAATTGTGA
2	HCN4	NM_005477.3	F: AATGAGGTGCTGGAGGAGTA R: TGGAGGAGGATGGAGTTCTT
3	HCN1	NM_021072.4	F: CATGCCACCGCTTTAATCCAG R: ATTGTAGCCACCAGTTTCCGA
4	HCN3	XM_011509817.2	F: AGCAGTGGAAATCGAGCAGG R: GGTCCCAGTAAAACCGGAAGT
5	KCCN4	NM_002250.3	F: CTGCTGCGTCTCTACCTGG R: AGGGTGCGTGTTCATGTAAAG
6	Cx30	NM_001110219.3	F: AAAGCAGAAGGTTCGGATAGAG R: GCAGGTGGTACCCATTGTAA
7	Cx40	NM_005266.7	F: GATTGGCCTGGTCTCTGTATTC R: GAACCTCCTTCTGAGCCTTTAC
8	Cx43	NM_000165.5	F: GGTGGTACTCAACAGCCTTATT R: CACCCGCTCATTCACATACA
9	B-actin	NM_001101.5	F: CACCATTGGCAATGAGCGGTTC R: AGGTCTTTGCGGATGTCCACGT

### ELISA

ELISA was used to determine the concentration of TBX3 and Cx30 proteins in the cells. The ELISA procedure used a Human TBX3 ELISA Kit (Finetest, EH1529) and Human Cx30 ELISA Kit (Finetest, EH8788) according to manufacturer's instructions. For intracellular protein measurement, the sample used was AD-MSCs lysate. Cell lysate sample preparation was carried out using the RIPA kit. For this, 0.5 ml of RIPA Lysis buffer (Thermo Scientific, 89900) was used for 2 × 106 cells and the DNA was removed first. Protein density was measured based on the absorbance produced at a wavelength of 450 nm with a microplate reader.

### Immunofluorescence

Cell samples from all treatment groups were fixed with 100% cold methanol for 5 min. Next, cells were washed using PBS and incubated with blocking buffer for 1 h. Cells were washed again and incubated at 4 °C with 5 µg TBX3 (Abcam, ab99302) and 5 µg Cx30 (Abcam, ab232456) primary antibodies overnight. Cells were washed and incubated at 4 °C with secondary antibody FITC (Abcam, ab6717) for TBX3 (1/1000) and Texas Red (Abcam, ab6787) for 1 h. After that, the samples were ready to be observed under a Nikon eclipse Ni fluorescence microscope.

### Patch clamp

Borosilicate glass microelectrodes used with tip resistances of 3–5 MΩ. Spontaneous action potentials were recorded using extracellular solutions (mM): NaCl 135, KCl 5.4, CaCl2 1.8, MgCl2 1.0, glucose 10, BaCl2 2.0, and HEPES 5.5 (pH 7.4, adjusted with NaOH). The pipette solution (mM): K + -ATP 110, KCl 20, CaCl2 5.0, MgCl2 5.0, HEPES 10, and EGTA 10 (pH 7.4, adjusted with KOH). Voltages are recorded with 5 kHz sampling rate. The examination was carried out using HEKA The EPC 10 double amplifier with software analysis Patchmaster v2 × 90.

### Statistical analysis

All quantitative data obtained from this study were presented in the form of average data ± standard error. qRT-PCR and ELISA data were analyzed by the One-Way ANOVA method. Each result was further tested using the Tukey Post hoc test method with a significance value of *p* < 0.05 to determine the significant differences between the data groups.

## Results

### MSC culture

**Fig. 1:**
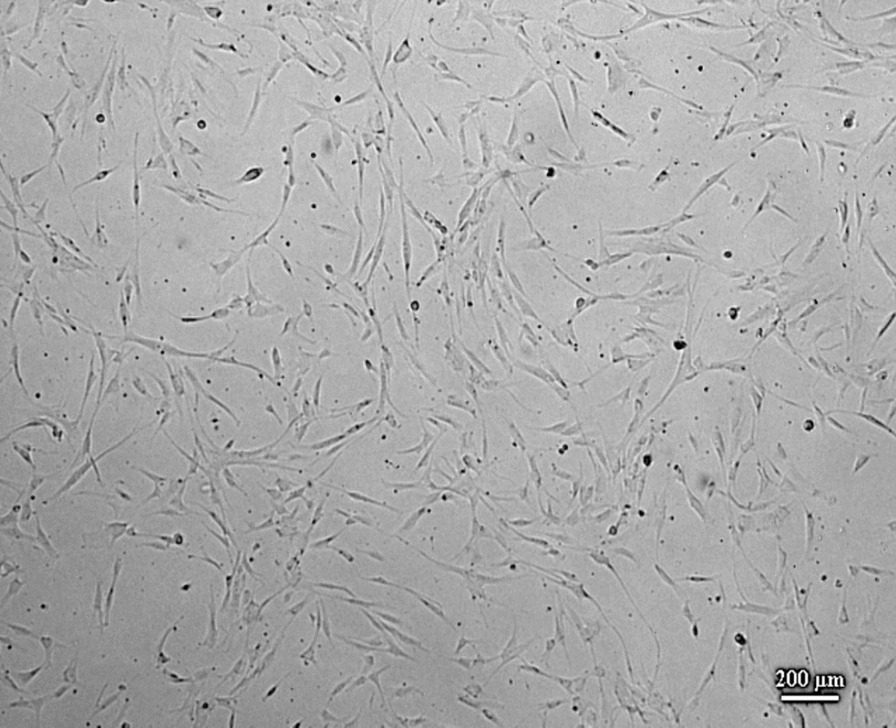
MSCs culture results from human adipose tissue with a confluency of 50% after 2 days of culture. The cells were planted in well plate 6. The microscope magnification was 40x

Figure 1 showed AD-MSCs morphology after 2 days of culture with confluencies ranging from 50% up to 60%. Based on the figure, it can be identified that AD-MSCs has shown its characteristics as MSCs, such as plastic adherent or can stick to the bottom of a plastic container and has an elongated and spiky morphology like fibroblast cells (fibroblast-like cells).

## MSC characterization using flowcytometry

**Fig. 2:**
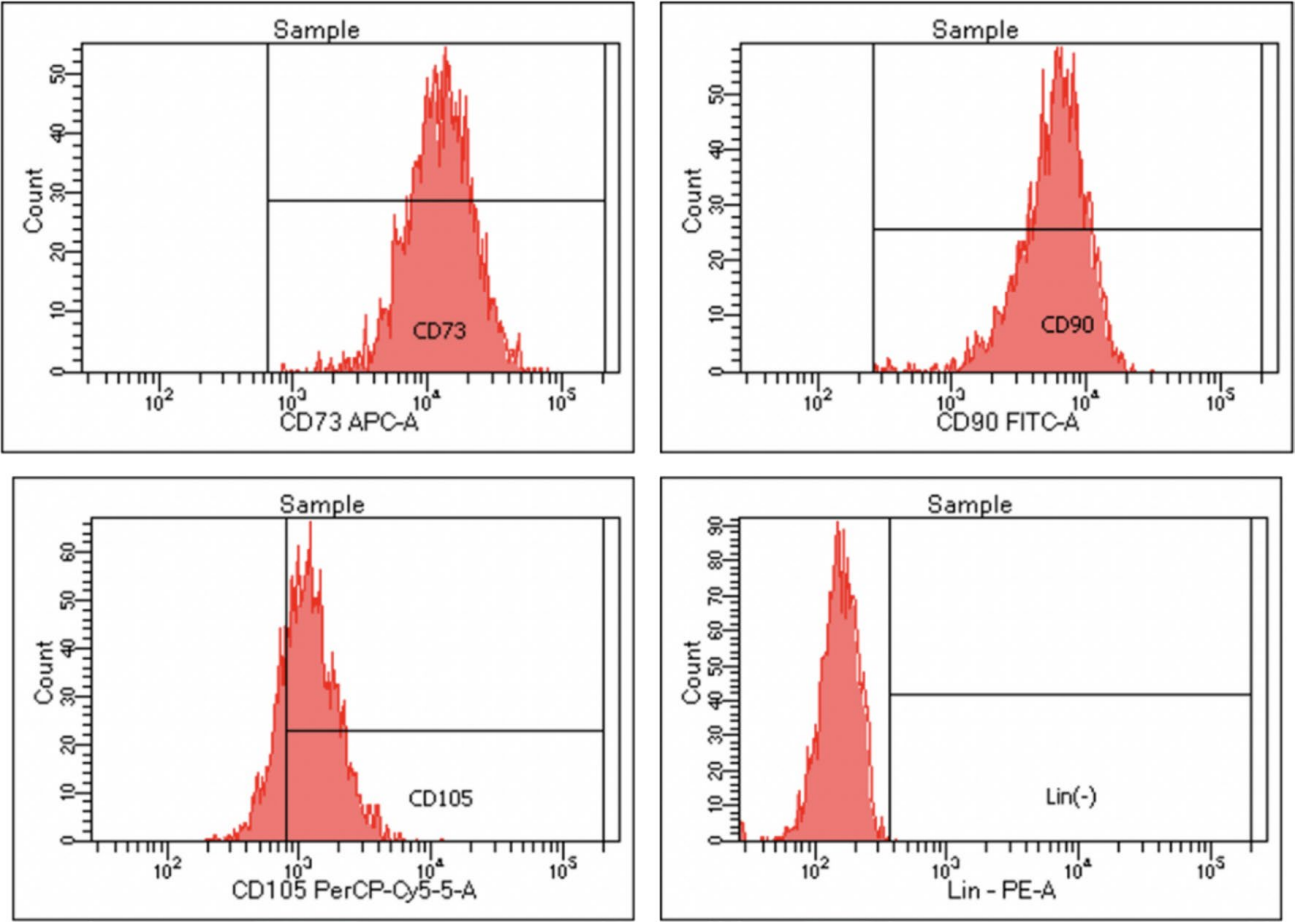
Flowcytometry results of AD-MSCs expressed 99.6% CD73, 99.4% CD90, and 98.5% CD105. The results also showed that < 2% of cells expressed negative markers of human MSC

Results are shown in Fig. 2. The AD-MSCs passage used for the experiment met the criteria as MSC. Cells used in the positive experiment expressed 99.4% CD90, 99.6% CD73, and 98.5% CD105. The results also showed that < 2% of cells expressed negative markers of human-MSC.

## MSC differentiation of the three lineages

**Fig. 3:**
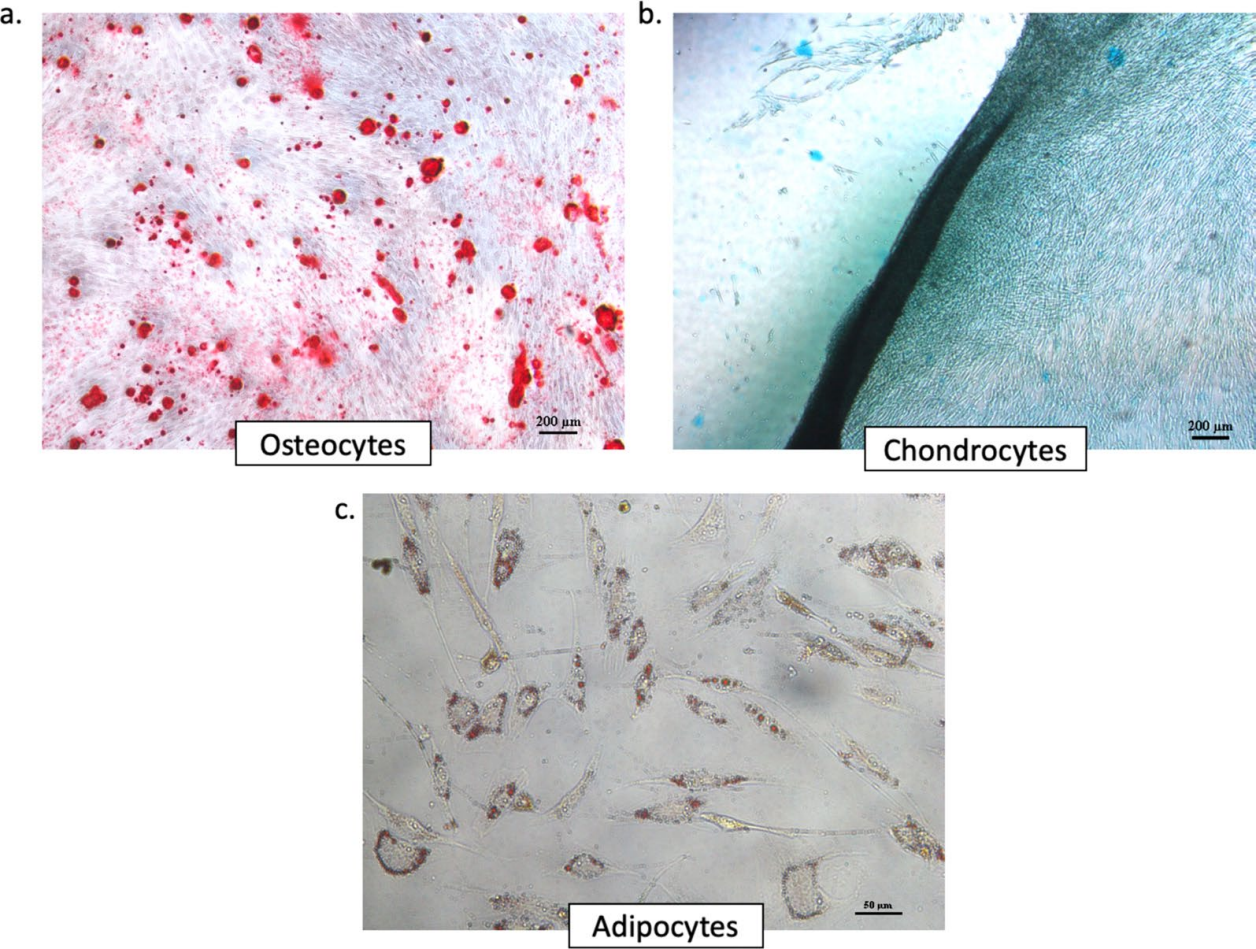
AD-MSCs showed differentiation into three lineages. **a** AD-MSCs have successfully differentiated into the osteogenic pathway, as indicated by cells that are colored red through binding to alizarin red dye. **b** AD-MSCs successfully differentiated into the chondrogenic pathway, indicated by the blue cells through binding to alcian blue dye. **c** AD-MSCs successfully differentiated into the adipogenic pathway, as indicated by the formation of fat “droplets” that bind to the oil red dye and show a red color

The results of cell staining for each differentiation pathway can be seen in Fig. 3. In Fig. 3a, MSCs have successfully differentiated into the osteogenic pathway, as indicated by cells that are colored red through binding to alizarin red dye. Figure 3b shows MSCs that have successfully differentiated into the chondrogenic pathway, indicated by the blue cells through binding to alcian blue dye. Figure 3c shows MSCs that have successfully differentiated into the adipogenic pathway, as indicated by the formation of fat “droplets” that bind to the oil red dye and show a red color. Based on the obtained results, it can be concluded that the stem cells used in this study met the differentiation criteria that must be possessed by MSC according to the ISCT agreement [21].

### qRT-PCR gene expression analysis

**Fig. 4:**
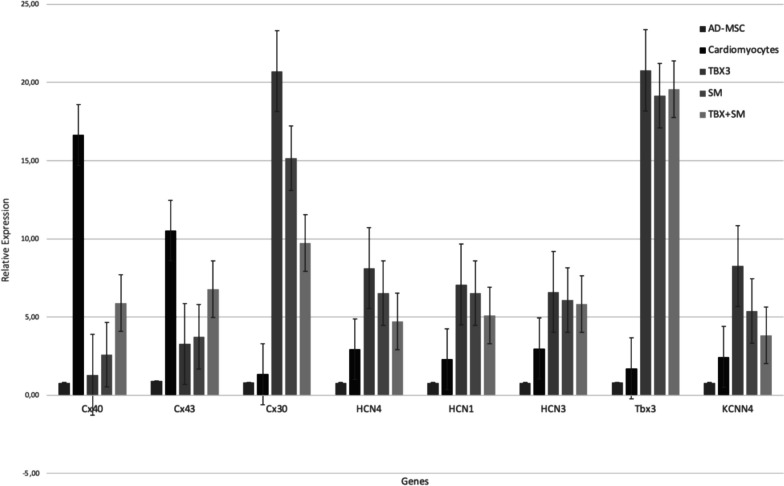
qRT-PCR gene expression analysis between AD-MSCs (control) group, cardiomyocytes group, TBX3 group, SM group, and TBX + SM group (n = 3)

The TBX3 gene expression in the control (x̄ = 1.00) and cardiomyocytes (x̄ = 1.73) group were small and not significantly difference (*p* = 0.999). In contrast TBX3 gene was expressed highest in the TBX3 group (x̄ = 20.54) compare to SM (x̄ = 18.53), and TBX + SM (x̄ = 17.18) groups. It’s favor that the transfected of TBX3 plasmid causes higher expression of TBX3 gene on cells. The TBX3 gene expression in all three treatment groups were significantly higher compare to the cardiomyocytes group (*p* < 0.001) (Fig. 4).

The Cx40 genes expression were significantly lower in TBX3 (x̄ = 1.95), SM (x̄ = 2.48), and TBX3 + SM (x̄ = 5.43) groups compare to cardiomyocytes group (x̄ = 15.27, *p* < 0.001). The Cx43 genes expression were significantly lower in TBX3 (x̄ = 3.04), SM (x̄ = 3.53), and TBX3 + SM (x̄ = 6.85) groups compare to cardiomyocytes group (x̄ = 11.62, *p* < 0.001). On the contrary, the Cx30 genes expression were significantly higher in TBX3 (x̄ = 22.19), SM (x̄ = 15.76), and TBX3 + SM (x̄ = 8.43) groups compare to cardiomyocytes group (x̄ = 1.31, *p* < 0.001).

The HCN1 genes expression were significantly higher in TBX3 (x̄ = 6.95), SM (x̄ = 6.25), and TBX3 + SM (x̄ = 5.37) groups compare to cardiomyocytes group (x̄ = 2.81, *p* < 0.001). The HCN3 genes expression were significantly higher in TBX3 (x̄ = 5.42), SM (x̄ = 5.97), TBX3 + SM (x̄ = 5.79) groups compare to cardiomyocytes group (x̄ = 2.19, *p* < 0.001). The HCN4 genes expression were significantly higher TBX3 (x̄ = 9.01), SM (x̄ = 6.22), TBX3 + SM (x̄ = 5.24) groups compare to cardiomyocytes group (x̄ = 2.10, *p* < 0.001). The KCNN4 genes expression were significantly higher TBX3 (x̄ = 8.26), SM (x̄ = 5.44), TBX3 + SM (x̄ = 4.81) groups compare to cardiomyocytes group (x̄ = 2.59, *p* < 0.001).

### ELISA

**Fig. 5:**
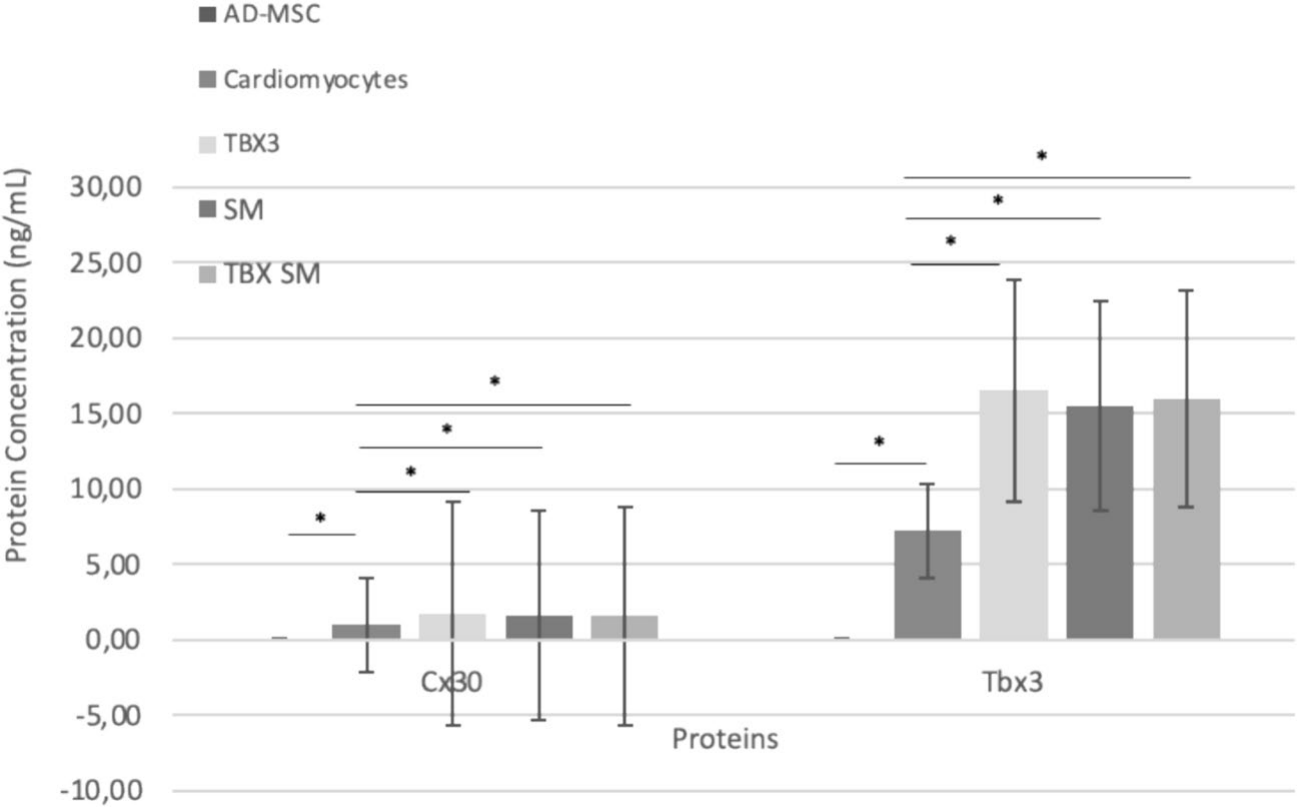
Effect of treatments with TBX3, SM, and the combination of both on the protein levels of Cx30 and TBX3 using ELISA analysis. Data showed overexpression of Cx30 and TBX3 protein in groups of treatments with TBX3, SM and TBX3 + SM as compared to cardiomyocytes group (*p* < 0.001). (TBX3: group that transfected with pcDNA TBX3, SM: group that added with small molecule SB431542, TBX3 + SM: Group that transfected with pcDNA TBX3 and added with with small molecule SB431542)

The results of ELISA analysis show higher concentration of TBX3 and Cx30 protein in TBX3, SM, and TBX3 + SM group compared to the control group and cardiomyocytes group (Fig. 5). The TBX3 protein in TBX3 (x̄ = 16.51 ng/mL), SM (x̄ = 15.49 ng/mL), and TBX3 + SM (x̄ = 15.99 ng/mL) groups showed significantly higher compared to cardiomyocyte group (x̄ = 7.25 ng/mL, *p* < 0.001). Same results showed in CX30 protein, that TBX3 (x̄ = 1.63 ng/mL), SM (x̄ = 1.59 ng/mL), and TBX3 + SM (x̄ = 1.52 ng/mL) groups showed a significant higher compared to cardiomyocyte group (x̄ = 1.01 ng/mL, *p* < 0.001).

### Morphology of AD-MSC

**Fig. 6:**
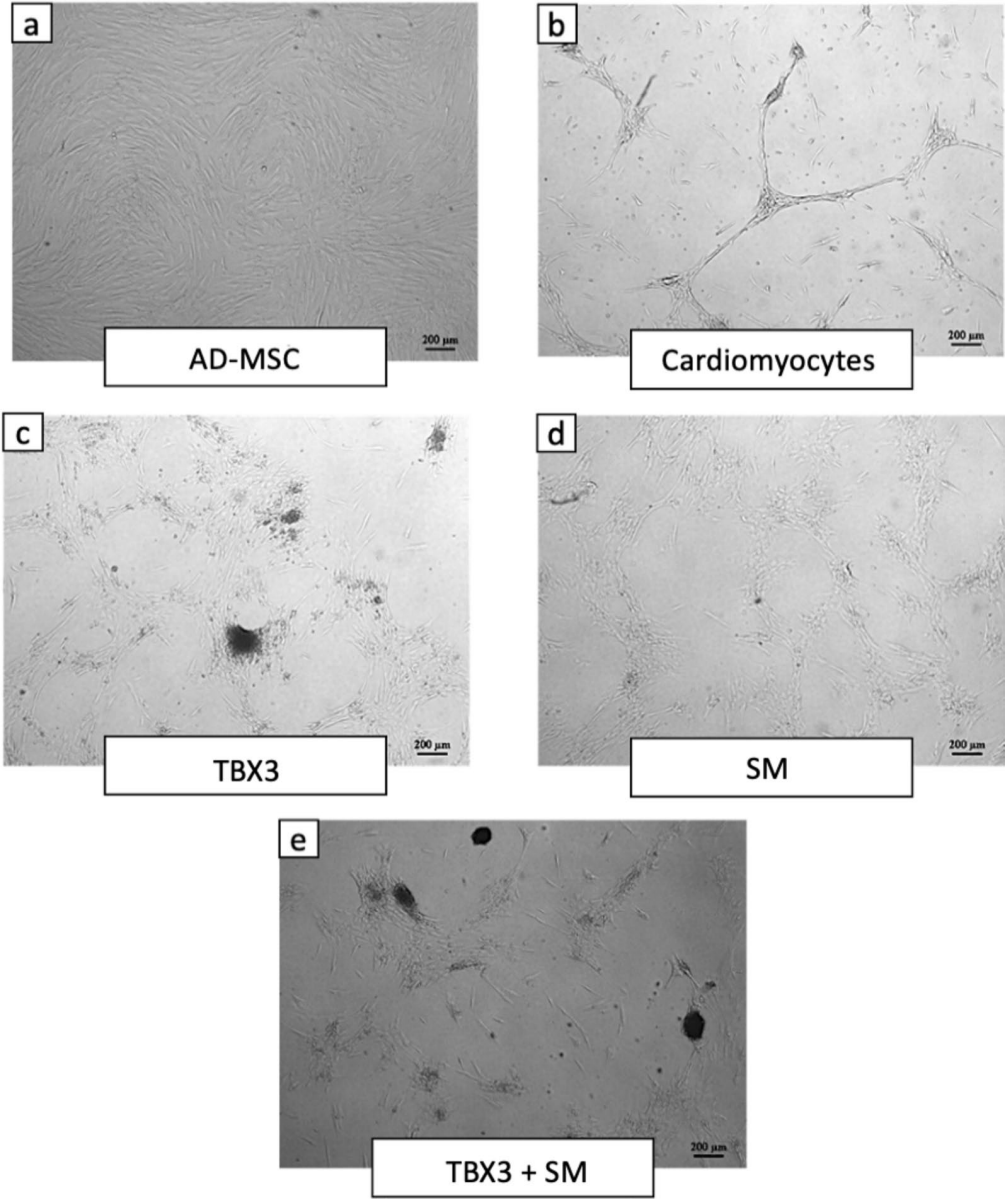
Morphology of the cells observed using invereted microscope at 40 × magnification. **a** Cells exhibit the characteristics of MSCs; they are elongated and pointed like fibroblast cells. **b** Cells gathered at one point and formed a new body, leaving empty places around it. **c** Cells morphology was also different from in the two previous groups, in this group, many black clusters of cells were found, which are a group of cells that formed black irregular circle body. These clusters of cells were also found in the TBX3 + SM group (**e**), and were not found in the SM group (**d**). (TBX3: group that transfected with pcDNA TBX3, SM: group that added with small molecule SB431542, TBX3 + SM: Group that transfected with pcDNA TBX3 and added with small molecule SB431542.)

Observation of the morphological results of AD-MSCs in all groups is presented in Fig. 6. The microscopic appearance of the AD-MSCs group is presented in Fig. 6a. The cells exhibit the characteristics of MSCs; they are elongated and pointed like fibroblast cells. Confluence in this group reached 100%. Figure 6b shows a group of AD-MSCs cells given cardiomyocyte differentiation medium. In this group, the cell morphology was different when compared to the AD-MSCs group. In the cardiomyocytes group, the cell distribution was not found to be 100% evenly distributed like AD-MSC group. On the contrary, in this group the cells gathered at one point and formed a new body, leaving empty places around it. In the group that was transfected with TBX3 (Fig. 6c), the cell morphology was also different from in the two previous groups. In this group, many clusters of cells were found, which are a group of cells that formed black irregular circle body. These clusters of cells were also found in the TBX3 + SM group (Fig. 6e) and were not found in the SM group (Fig. 6d).

### Immunofluorescence

**Fig. 7:**
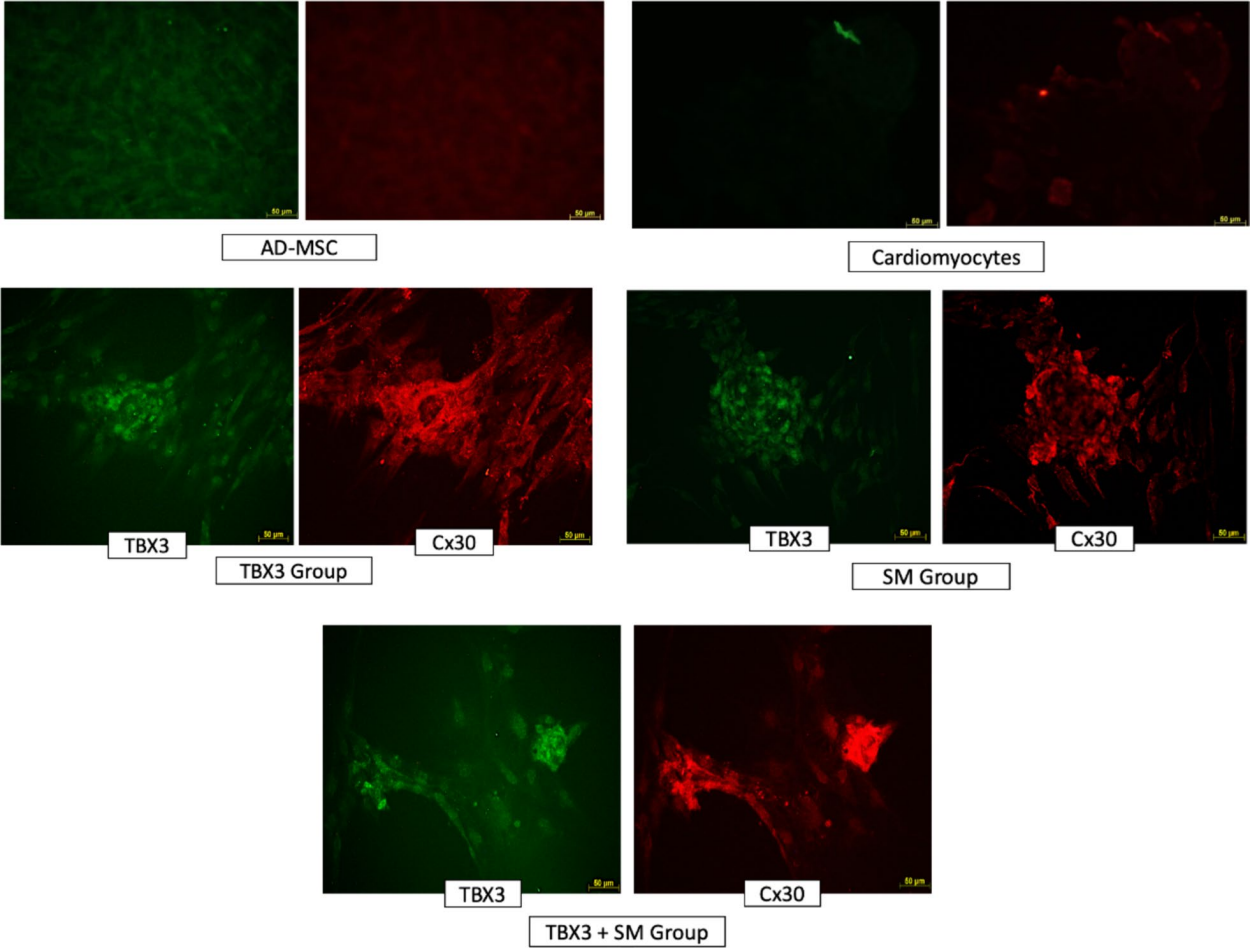
Result of immunofluorescence. It showed the green glow for the appearance of the TBX3 protein, and red glow for the appearance of Cx30 protein. Expression of TBX3 and Cx30 proteins showed in TBX3, SM, and TBX3 + SM group. The AD-MSCs and cardiomyocyte group did not show a positive glow. (TBX3: group that transfected with pcDNA TBX3, SM: group that added with small molecule SB431542, TBX3 + SM: Group that transfected with pcDNA TBX3 and added with with small molecule SB431542.)

In the immunofluorescence observation, TBX3 groups had positive TBX3 signal as indicated by green glow and positive Cx30 indicated by red glow for positive TBX3. The AD-MSCs and cardiomyocyte group did not show a positive glow (Fig. 7).

### Patch clamp

Electrophysiological function of cells were tested using patch clamp with a whole-cell configuration to allow measurement of the action potential across the entire cell. Two examinations were carried out such as spontaneous action potential (AP). Analysis of the AP was carried out AP morphology, ratio of AP duration at 90% repolarization (APD90), ratio of AP duration at 50% repolarization (APD50), and the ratio of APD90 to APD50 ratio (APD90/APD50). When APD90/APD50 has a number < 1.4, it shows AP of ventricular cells, number 1.4 to 1.7 shows AP of nodal or pacemaker cells, while number > 1.7 shows AP of atrial cells [22].

**Fig. 8:**
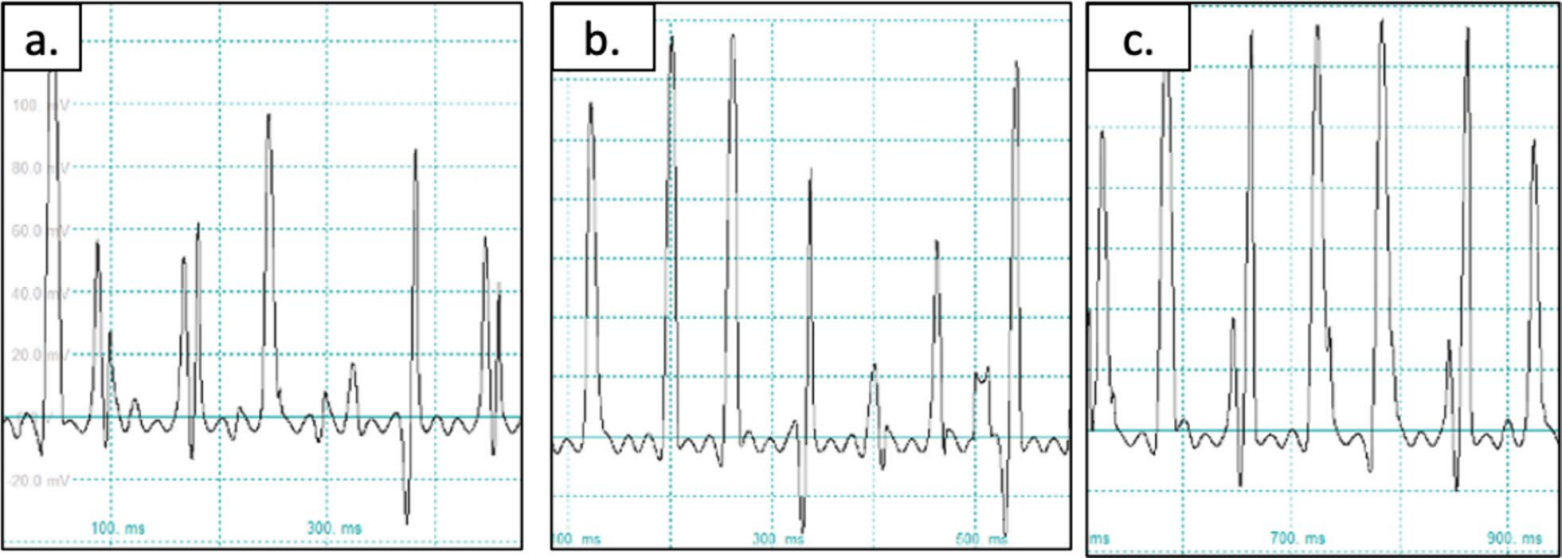
Spontaneous action potential morphology in **a** TBX3 group, **b** SM group, **c** TBX3 + SM group

**Fig. 9:**
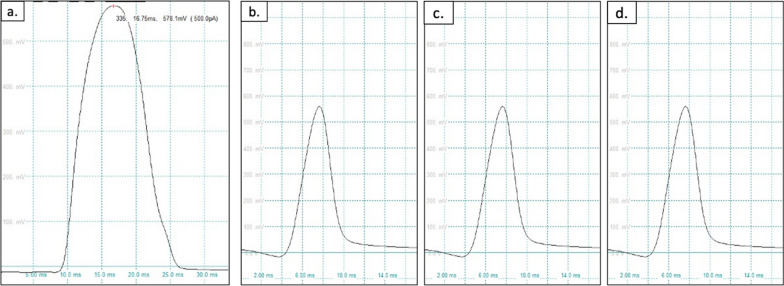
Action potential features in **a** cardiomyocyte group (atrial cell morphology), **b** TBX3 group (pacemaker morphology), **c** SM group (pacemaker morphology), **d** TBX3 + SM (pacemaker morphology)

In TBX3, SM, and TBX3 + SM group, spontaneous action potential morphology was obtained (Fig. 8a–c) as well as action potential features showing the characteristics of pacemaker cells (Fig. 9b–d). As for the cardiomyocyte group, there was no spontaneous action potential and action potential features showing the characteristics of atrial cell (Fig. 9a).

The analysis of the ratio of the duration of APD90/APD50 also supports these morphological findings. It can be seen in Table 2, where the TBX3 group obtained a ratio of 1.62 ± 0.50, SM group obtained a ratio of 1.54 ± 0.03, and TBX3 + SM obtained a ratio of 1.60 ± 0.20. In contrast to the cardiomyocyte group the result was 1.91 ± 0.05.

**Table 2:** Average of action potential duration value (x̄ ± SD)

Group	APD90/APD50	Type of cell
Cardiomyocyte	1.91 ± 0.05	Atrial-like cell
TBX3	1.62 ± 0.50	Pacemaker-like cell
SM	1.54 ± 0.03	Pacemaker-like cell
TBX + SM	1.60 ± 0.20	Pacemaker-like cell

## Discussion

The main finding of our study was that the transfection of the TBX3 plasmid and the addition of the small molecule SB431542 can lead to the differentiation of AD-MSCs into CPLCs, either given individually or combined with both of them. But in this study, the best results were shown in the group transfected with plasmid TBX3. Compared to the SM and TBX3 + SM groups, treatment using only TBX3 gene transfection resulted in cells expressing higher levels of the gene and protein markers of CPLCs. Gene transfection works by inserting a plasmid, TBX3, into the nucleus with the help of lipofectamine. Transfection of the plasmid was conducted on day 3 of 15-day differentiation, the stage before MSCs committed to becoming cardiac progenitor cells. MSC differentiation into cardiomyocytes has a shorter stage due to their nature as multipotent cells. Cardiac differentiation of MSCs starts with multipotent cells (day 0–4), cardiac progenitor cells (day 5–6), immature cardiomyocytes (day 7), and mature cardiomyocytes (after day 7) [23].

TBX3 is specifically expressed in the atrioventricular (AV) conduction system, and loss-of-function and gain-of-function experiments have demonstrated that TBX3 is required for cardiac conduction system development and homeostasis [24]. Study conducted by Zhao in 2020 [25], in which overexpression of the TBX3 gene was found to induce a reduction in the expression profile of working cardiomyocytes to become pacemaker cells. Vedantham [26] also described the function of TBX3 as a transcriptional repressor in pacemaker cells by silencing the expression of genes associated with force-generating cardiomyocytes and indirectly promoting the expression of genes in the pacemaker program. The TBX3 gene can also reduce intercellular coupling, reduce LK1 density to activate cardiac diastolic depolarization, and reprogram working cardiomyocytes [10]. Mohan et al. explored whether the AV conduction system is affected by TBX3 dose reduction through the characterization of electrophysiological properties and morphology of heterozygous TBX3 mutant (TBX3^+/−^) mouse hearts. They found PR interval shortening and prolonged QRS duration, as well as atrioventricular bundle hypoplasia after birth in heterozygous mice. The TBX3^+/−^ AV nodes showed increased expression of working myocardial gene programs (mitochondrial and metabolic processes, muscle contractility) and reduced expression of pacemaker gene programs (neuronal, Wnt signaling, calcium/ion channel activity). Furthermore, they identified TBX3-dependent regulatory DNA elements active in the AV conduction system and validated the functionality of these elements. Deletion of a regulatory DNA elements drives expression of Cacna1g in the cardiac conduction system [27].

TBX3 not only suppresses differentiation but also induces the action of the HCN family, Cx45 and Cx30, and suppresses the action of Cx40 and Cx43 [10]. The results of the qRT-PCR analysis carried out on connexin genes such as Cx30, Cx40, and Cx43 showed the same results as research conducted by Zhao in 2020 [28], which demonstrated an increase in the expression of the Cx30 gene as well as a decrease in Cx40 and Cx43 gene expression. Cx30 contributes to the slowing of impulse propagation from the AV node and limits the maximum heart rate carried from the atria to the ventricles, thereby preventing rapid conduction of potentially worsening hemodynamics to the ventricles [26, 28]. In addition to this, Cx30 also integrates all cardiac pacemaker cells with different intrinsic frequencies [29].

HCN4 is the most expressed ion channel in the SA node. During development, HCN4 is initiated in the cardiac crescent and is progressively reduced and retained in the SA node during differentiation and in the adult heart. Inhibition of HCN4 regulation leads to lower expression of TBX3, SHOX2, BMP4, and Cacna1g in SA node development. This proves that an increase in TBX3 will affect HCN4 in the SA node, accompanied by the pacemaker phenotype that is still maintained [30]. In addition, cultured embryonic hearts of the transgenic mouse line which were treated with Bmp2, resulted in induction of endogenous AV canal genes (Tbx2, Tbx3) and reduction of chamber myocardium markers (Nppa, Nppb) [31]. The increase in HCN1 and HCN3 expression in our study can be explained by Ragunathan’s study [15], which revealed that when HCN4 is highly expressed by TBX3, HCN1 and HCN3 will be highly expressed as well [32].

The Ca^2+^ ions play a role in the regulation, flexibility, and contractility of the heart. The entry of Ca^2+^ ions through the channel not only plays a role in initiating cardiac excitation and contraction but also affects several crossing pathways that can change the membrane potential [29]. KCNN is an ion channel that plays an important role in Ca^2+^ exchange and the regulation of cardiac excitability [32]. One of the sub-families of KCNN is KCNN4. KCNN4 is an intermediate type of calcium-activated potassium channel that plays an important role in functional activity in the pacemaker [30]. Study conducted by Kleger [33], which showed an increase in the expression of Cx30 and KCNN4 in pacemaker-like cells. Raghunathan [15] also noted that there was an increase in the expression of specific pacemaker-like cell genes, including TBX3, KCNN4, Cx30, and BMP2.

Nodal inhibitors work by inhibiting the nodal protein, which is a protein that is secreted and binds to membrane proteins such as serine/threonine kinase receptors type I and II [34]. After activating the receptor, signal induction is then continued by the Smad2/3 protein, which in turn activates gene transcription along with the FoxH1 and Smad4 proteins, transcribing the nodal gene itself, the Lefty gene, and the PITX2 gene [35]. PITX2 protein activity can reduce the expression of the SHOX2 transcription factor, which plays a role in increasing TBX3 gene expression. From this pathway, we can see that the effect of adding nodal protein inhibitors does not directly increase TBX3 gene expression. It's different if we use gene transfection treatments, which have a direct effect on increasing TBX3 gene expression.

The combination of nodal inhibitor and TBX3 transfection did not show better results; it is also possible because of the accumulation of nodal inhibitor concentrations that were given continuously during the time of differentiation. This makes the cell viability decrease, as happened in the first batch. However, the results of the three treatment groups showed positive differentiation of MSCs into pacemaker-like cells. So, it is possible that adding small molecules is sufficient to increase TBX3 gene expression and differentiation of MSCs into pacemaker-like cells, although by transfecting the gene, the results of TBX3 gene expression were higher than other methods.

Electrophysiological examination using a patch clamp was also carried out to prove there were differences in the differentiation of the three treatment groups compared to the cardiomyocyte group. This difference was evident from the absence of spontaneous action potentials in the cardiomyocyte group, whereas in the TBX3, SM and TBX3 + SM groups, spontaneous action potentials were seen. This is similar to the study by Zhao et al. [28] that transfected TBX3 to iPSCs, also study by Sergei Yechikov et al. [5] that addded small molecules to iPSCs. From the morphological analysis of the stimulated action potential, it can be seen that the morphology of the action potential of the cardiomyocyte group looks more like an atrial action potential while in the treatment groups looks more like a pacemaker cell action potential. This is also in line by comparing the ratio of APD90 to APD50 where it was found in the cardiomyocyte group that the average APD90/APD50 ratio was 1.91 ± 0.05 which according to Rajamohan et al. [22] is the ratio of atrial action potentials, while in the treatment group the ratio of APD90/APD50 between 1.4 and 1.7 which is the ratio of the action potential of the pacemaker cells. Thus the results of the electrophysiological examination can provide support from the results of genetic expression examinations and protein analysis that there has been a change in the differentiation process from MSCs to typical cardiomyocytes of pacemaker cells in TBX3, SM, and TBX3 + SM group.

### Limitations

The study has limitation as we used single donor of AD-MSCs obtained from liposuction which was a stored biological material in the Stem Cells and Tissue Engineering (SCTE) laboratory, IMERI, Faculty of Medicine, University of Indonesia. The use of one donor sample in this study was by intention to minimize variability in this initial study and to save the cost. Glass et al. [36] studied age effect on gene expression by examining expression profiles in adipose tissue from 856 female twins aged from 39 to 85 years old. They found that the genes had lower levels of expression (50.8%) with age in adipose tissue. It was why we chose 19 years old healthy female as the source of AD-MSCs. However, the use of single cells sample is still sufficient for studies of gene expression differentiation [37]. Yet, we successfully produce high purity MSCs.

In conclusion, this study indicated that transfection of transcription factor TBX3 is capable to initiate differentiation of human AD-MSCs into CPLCs.

### Potential clinical application

The first step, we currently are developing a bigger animal model with complete heart block that mimicking degenerative process. The animal model will serve as recipient of our CPLCs product. Dose effect correlation will be studied. Secondly, phase one clinical trial in limited subjects with partially dependent to pacemaker device will give important information of any AV conduction improvement. In the future, the cells might be used as a therapy to correct AV conduction abnormalities.

## Data Availability

The data is not publicly available. The corresponding author Yoga Yuniadi (yogay136@gmail.com) can be contacted to request permission to view the data.

## Abbreviations

Ad-MSC’s: Adipose mesenchymal stem cells
ANF: Atrial natriuretic factor/peptide
AP: Action potential
AV: Atrio ventricle
AVC: Atrio ventricle cannal
AVN: Atrioventricular node
bFGF: Basic fibroblast growth factor
BMP: Bone morphogenetic protein
BMSC: Bone marrow stem cells
CT: Cycle threshold
CVD: Cardiovascular disease
Cx: Connexin
DNA: Deoxyribonucleid acid
ELISA: Enzyme linked immunosorbent assay
ESC: Embryonic stem cells
GVHD: Graft versus host disease
HB: His bundle
HCN: Hyperpolarization activated cyclic nucleotide gated potassium channel
HGF: Hepatocyte growth factor
HSC: Hematopoietic stem cell
Id-2: Inhibitor of DNA binding-2
IF: Immunofluorescence
IGF-1: Insulin-like growth factor
ISL-1: Insuline gene enhancer binding protein-1
KCNN: Potassium calcium-activated channel subfamily n member
LSH: Left-Sinous Horn
MCP-1: Monocyte chemotactic protein
MI: Myocardial infarction
mRNA: Messenger RNA
MSC's: Mesenchymal stem cells
NLA: Nuclear localization signal
pcDNA: Plasmid cloning DNA
Pitx2: Paired-like homeodomain transcription factor 2
PPM: Permanent pacemaker
qRT-PCR: Real-Time Quantitative Reverse Transcription PCR
RNA: Ribonucleid acid
SAN: Sinoatrial node
SHOX2: Short Stature Homeobox 2
SSS: Sick sinus syndrome
TBX3: T-Box Transcription Factor 3
TGF-β: Transforming growth factor-β
